# A single centre study of 41 cases on the use of porous tantalum metal implants in acetabular revision surgery

**DOI:** 10.1186/s12891-019-2626-9

**Published:** 2019-05-22

**Authors:** Christoph Theil, Tom Schmidt-Braekling, Georg Gosheger, Burkhard Moellenbeck, Jan Schwarze, Ralf Dieckmann

**Affiliations:** Department of Orthopaedics and Tumour Orthopaedics, Muenster University Hospital, Muenster, Albert-Schweitzer-Campus 1, 48149 Muenster, Germany

**Keywords:** Periprosthetic infection, Acetabular revision, Megaprostheses, Two-stage revision, Revision arthroplasty

## Abstract

**Background:**

This study aims at investigating cup survival of porous tantalum revision cups and identifies risk factors for failure.

**Methods:**

We retrospectively reviewed 41 patients treated between 2010 and 2012. Main indications were aseptic loosening in 83% and two-stage exchange after periprosthetic joint infection in 17% of cases. Mean follow-up period was 72 months. Femoral megaprostheses were used in 13% of cases. Most defects were classified as Paprosky 3b (29%). Function was assessed using the Harris Hip score.

**Results:**

Aseptic cup survivorship was 80% at 104 months (95% Confidence Interval 67.4–92.4). Overall implant survival was 73%. Major bone loss defects (Paprosky types 2c to 3b) were associated with a significantly higher rate of failure than minor defects (*P* = 0.002). There were eight cases of aseptic loosening (19.5%) and two of infection (4.9%). Previous surgeries, indication for acetabular revision, patient-related risk factors and use of megaprostheses did not significantly influence implant survival. The Harris Hip Score improved from a median of 40 (Interquartile range 31–45) to 82 (interquartile range 65–88) postoperative (*P* < 0.0001).

**Conclusions:**

In summary, the use of porous tantalum metal implants in acetabular revision surgery achieves good to excellent short- term and mid-term functional results and an acceptable complication rate relative to the extent of defect and previous surgery. However, one should be aware of potential limitations of the implants in addressing large defects and discontinuity.

## Background

Managing acetabular defects in total hip revision arthroplasty is a challenge for orthopaedic surgeons and demands careful implant selection. An ideal implant should provide immediate primary stability and long term bony ingrowth with reconstruction of hip anatomy and biomechanics. There have been many different treatment options as described in a recent review [1]. For smaller contained defects cementless revision cups in combination with morcellised bone graft provide good long-term results [2, 3]. For larger defects the use of structured bone grafts [4] has been well established and jumbo cups [5] have provided excellent clinical results with > 90% long term implant survival. Another common treatment option is the use of an antiprotrusion cage like the Burch-Schneider cage that provides good results [6, 7] in combination with cemented cups and bone graft.

Highly porous metals like trabecular metal made out of porous tantalum have several advantages over conventional cementless cups: higher volume porosity, freely communicating pores, a higher coefficient of friction against bone and lower bulk stiffness so that bony ingrowth is facilitated while preserving sufficient initial stability without risk for resorption [8].

The aim of this study is to assess the outcome after trabecular metal reconstruction for acetabular defects and to discuss potential risk factors for failures with particular attention given to defect size and location.

## Methods

Ethical approval was obtained prior to conducting the study by the local institutional review board (No. 2017–209-f-S). Trabecular metal implants were widely used in our department by a single manufacturer between 2010 and 2012 for acetabular reconstruction.

A review of the institution’s database was carried out to identify patients who underwent acetabular component revision between January 1, 2010 and December 31, 2012. Patients who underwent revision procedures without the use of trabecular metal porous metal cup components, or who were treated for an oncologic indication, or who had a follow-up period of less than 2 years were excluded. A total of 41 patients were identified using these criteria. The clinical course of treatment, and failures that occurred during the follow-up period, were recorded.

### Definitions and surgical procedures

Aseptic loosening was diagnosed on the basis of clinical and radiological findings. A previous joint aspiration was carried out beforehand in all patients. Periprosthetic joint infection (PJI) was diagnosed using the Musculoskeletal Infection Society (MSIS) criteria [9] and treated using a two-stage revision protocol with a minimum of 6 weeks of antibiotics between stages. Postoperative treatment was standardized with partial weight bearing for 6 weeks to allow for osseous integration. Standard hip precautions to prevent dislocation were applied.

Previous surgery was defined as an exchange of the acetabular cup prior to the implantation of a trabecular metal cup. The patient’s previous medical and surgical history was assessed to calculate the Charlson comorbidity score at the time of acetabular revision [10].

During the follow- up period, patients were generally examined radiologically and clinically after 3 months 3 months and then annually in our outpatient clinic. Harris hip score [11] was obtained at the last follow-up visit for patients with retained acetabular trabecular metal implant. Patients with implant failure prior to completion of the rehabilitation who underwent early revision surgery were not included in the assessment of functional results. Anteroposterior pelvic radiographs were used to measure the vertical center of hip rotation [12, 13]. A high hip center was defined as being at least 35 mm above the interteardrop line [14]. Defects were classified in accordance with the Paprosky classification [15] based on radiographic findings(conventional radiographs and computed tomography scans when available) and intraoperative description of the acetabular defect by one senior orthopaedic surgeon (Table 1). Acetabular bone loss was defined as major in Paprosky defects type 2c to 3b and minor in 2a and 2b defects [16].

**Table 1:** patient demographics and surgical factors

Patient and surgical factors	results
median age in years(IQR)	66 (58–72)
median BMI in kg/m^2^(IQR)	27 (26–31)
median number of previous surgeries for PJI(IQR)	0 (0–1)
median numbers of previous aseptic acetabular revisions(IQR)	0 (0–3)
median Charlson comorbidity index(IQR)	3 (2–5)
indication for acetabular revision	
aseptic loosening	83%
reimplantation after two-stage exchange	17%
median follow-up (IQR)	72 (42–87)
femoral megaprosthetic reconstruction %	13%
total femoral replacement	5%
proximal femoral replacement	8%
Paprosky classification of acetabular defect	
2a	24%
2b	17%
2c	15%
3a	15%
3b	29%
Major bone loss	69%
Minor bone loss	31%

Trabecular metal™(TM, Zimmer, Inc., Warsaw, IN, USA) implants were used for aseptic revisions and non-resistant bacteria a clindamycin and gentamicin PMMA cement was used (Copal G + C™, Heraeus GmbH, Berlin, Germany); otherwise vancomycin cement was used(Copal G + V™, Heraeus GmbH, Berlin, Germany). Femoral megaprosthetic reconstruction when necessary was performed using the MUTARS™ – modular universal tumour and revision system (Implantcast GmbH, Buxtehude, Germany). Impaction bone grafting using autograft, allograft bone (Tutogen™, RTI Corp., Alachua, FL, USA) or bone substitute (Vitoss, Stryker Corp., Kalamazoo, MI, USA).

The primary endpoint was aseptic cup survival resulting in revision. Secondary endpoints were loss of implant for other reasons, and death. In our study the only two reasons for implant failure were aseptic loosening defined as failing Moore’s criteria [17] accompanied by clinical symptoms and periprosthetic joint infection which was diagnosed following MSIS criteria [9] and was treated with a two-stage revision protocol.

### Statistical analysis

Data collection and statistical analysis were carried out using Excel (Microsoft Corporation, Redmont, Washington, USA) and SPSS Version 25(IBM Corporation, Armonk, NY, USA). Survival was evaluated using Kaplan-Meier curves and log rank test for differences in survival. Descriptive statistics and Shapiro-Wilk test were used to determine distribution of data. Crosstables, Fisher’s test and Chi^2^ test were used to determine correlation between categorical variables. Statistical significance was defined as *p* ≤ 0.05, 95% confidence intervals (CI) are provided. For non-parametric data comparative statistical analysis was done using the Wilcoxon rank test and Mann-Whitney U-test.

## Results

The demographic data and surgical details for the patients are shown in Table 1.

The revision-free survival rate of trabecular metal acetabular components was 73% at 104 months (95% CI 59 to 86%). There were a total of 10 failures, eight of which occurred during the first year after surgery. All of the failures resulted in the loss of the acetabular component. Table 2 lists details of all of the cases.

**Table 2:** patient and surgery details

Number	Age at surgery	CCI	Previous surgeries	Indication	TM components	Megaprosthesis?	Defect classification	Failure mode	Final implant	Follow up
1	67	2	2	Aseptic loosening	Acetabular shell	no	2a	none		53
2	46	0	2	Aseptic loosening	Acetabular shell	no	2b	none		45
3	79	4	2	Aseptic loosening	Acetabular shell	no	2b	none		56
4	32	0	2	Aseptic loosening	Acetabular shell and wedges	no	3b	none		56
5	76	9	0	Aseptic loosening	Acetabular shell and wedges	no	3b	Aseptic loosening	Antiprotrusio cage, dual-mobility cup	46
6	79	9	0	Aseptic loosening	Acetabular shell	no	2a	none		50
7	56	2	0	Aseptic loosening	Acetabular shell and wedges	no	3a	none		23
8	73	9	0	Aseptic loosening	Acetabular shell	no	2a	none		90
9	71	2	2	Aseptic loosening	Acetabular shell	no	2a	none		89
10	73	3	3	Aseptic loosening	Acetabular shell	no	2c	PJI	disarticulation	7
11	71	2	2	Aseptic loosening	Acetabular shell	Yes, total femur	2b	none		29
12	50	8	2	Aseptic loosening	Acetabular shell and wedges	no	3b	Aseptic loosening	Antiprotrusio cage, dual-mobility cup	87
13	69	2	3	Aseptic loosening	Acetabular shell and wedges	no	3b	Aseptic loosening	Antiprotrusio cage, dual-mobility cup	33
14	69	2	2	Aseptic loosening	Acetabular shell	no	2a	none		37
15	61	1	3	Aseptic loosening	Acetabular shell, cup-cage	no	3b	none		84
16	67	2	3	Periprosthetic joint infection	Acetabular shell	no	2b	none		41
17	56	1	2	Aseptic loosening	Acetabular shell, wedge	no	3a	none		83
18	50	1	2	Periprosthetic joint infection	Acetabular shell, cup-cage	no	3a	none		83
19	42	4	3	Periprosthetic joint infection	Acetabular shell	no	2b	none		83
20	82	3	3	Periprosthetic joint infection	Acetabular shell, wedges, cup-cage	Yes, proximal femur	3b	death		7
21	59	1	3	Aseptic loosening	Acetabular shell, wedges	no	3b	Aseptic loosening	Antiprotrusio cage, dual-mobility cup	79
22	61	6	2	Periprosthetic joint infection	Acetabular shell	no	2c	none		74
23	66	3	1	Aseptic loosening	Acetabular shell	no	2a	none		74
24	59	1	2	Aseptic loosening	Acetabular shell, wedges, buttess	Yes, proximal femur	3b	none		70
25	61	2	5	Aseptic loosening	Acetabular shell, wedge	no	3a	none		98
26	65	2	2	Aseptic loosening	Acetabular shell, wedges	no	3b	Aseptic loosening		96
27	63	1	3	Aseptic loosening	Acetabular shell, wedge	no	2c	none	Bipolar head	95
28	57	3	2	Aseptic loosening	Acetabular shell	no	2c	Aseptic loosening	Antiprotrusio cage, dual-mobility cup	25
29	57	2	0	Aseptic loosening	Acetabular shell	no	2c	none		87
30	78	9	0	Aseptic loosening	Acetabular shell	no	2b	none		28
31	80	10	1	Aseptic loosening	Acetabular shell	no	2b	none		78
32	74	3	0	Aseptic loosening	Acetabular shell	no	2a	none		52
33	58	1	0	Aseptic loosening	Acetabular shell	no	2a	none		75
34	62	5	4	Aseptic loosening	Acetabular shell	no	2a	none		70
35	72	4	3	Periprosthetic joint infection	Acetabular shell	Yes, proximal femur	2a	none		104
36	76	7	4	Periprosthetic joint infection	Acetabular shell, cup-cage	Yes, total femur	3a	PJI	Antiprotrusio cage, dual-mobility cup, total femur	57
37	62	4	3	Aseptic loosening	Acetabular shell	no	2c	Aseptic loosening	Antiprotrusio cage, dual-mobility cup	98
38	72	4	0	Aseptic loosening	Acetabular shell, wedge, cup-cage	no	3b	none		26
39	69	4	2	Aseptic loosening	Acetabular shell, wedge	no	3a	Aseptic loosening	Antiprotrusio cage, dual-mobility cup	104
40	69	4	3	Aseptic loosening	Acetabular shell, wedge, cup-cage	no	3b	none		103
41	76	7	0	Aseptic loosening	Acetabular shell, cup-cage	no	3b	none		26

There were eight cases of aseptic loosenings (19.5%) and 2 infections (4.9%). One infection occurred after 45 months in a patient with a total femur replacement after multiple femoral revisions and was treated with two-stage revision. One other patient suffered from early PJI and was treated with debridement, exchange of mobile parts and antibiotics at first then underwent explantation of the prosthesis and finally hip disarticulation when infection persisted. In patients treated for PJI and underwent reimplantation with a trabecular metal acetabular component, infection was eradicated in 83% of all cases with one case of recurrent infection, as mentioned above.

Five patients who suffered from early loosening had a Paprosky 3b defect. Two of these patients presented with pelvic discontinuity, two had severe pelvic metallosis after femoral cone fracture and delayed diagnosis and one had primary total hip arthroplasty for hip dysplasia. The patients with aseptic loosening were all revised and treated with an antiprotrusion ring reconstruction with a dual mobility cup. One case of pelvic discontinuity was treated with large bipolar head and acetabular component removal.

**Fig. 1:**
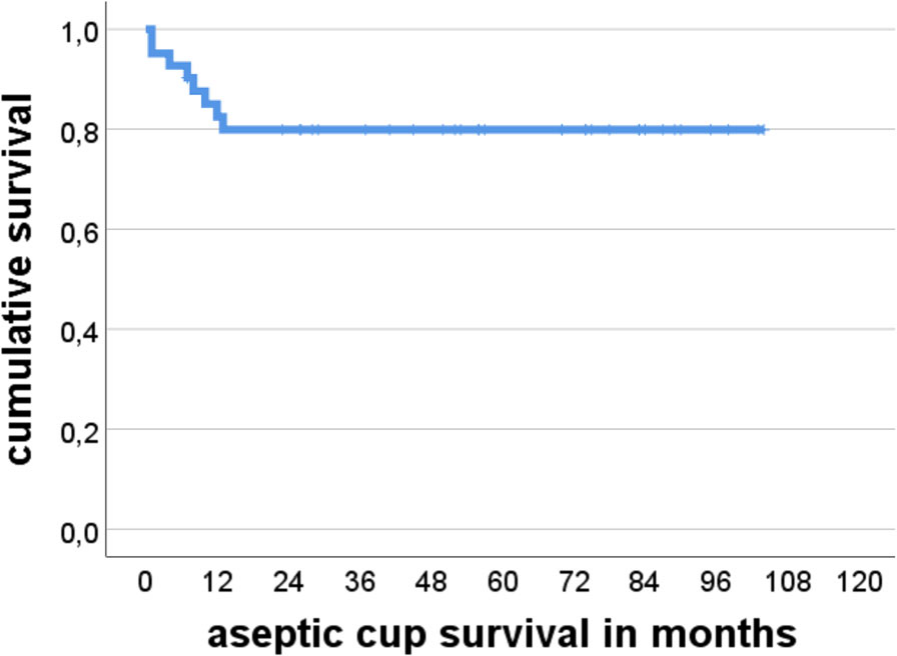
Cumulative Kaplan–Meier aseptic cup survival in months

**Fig. 2:**
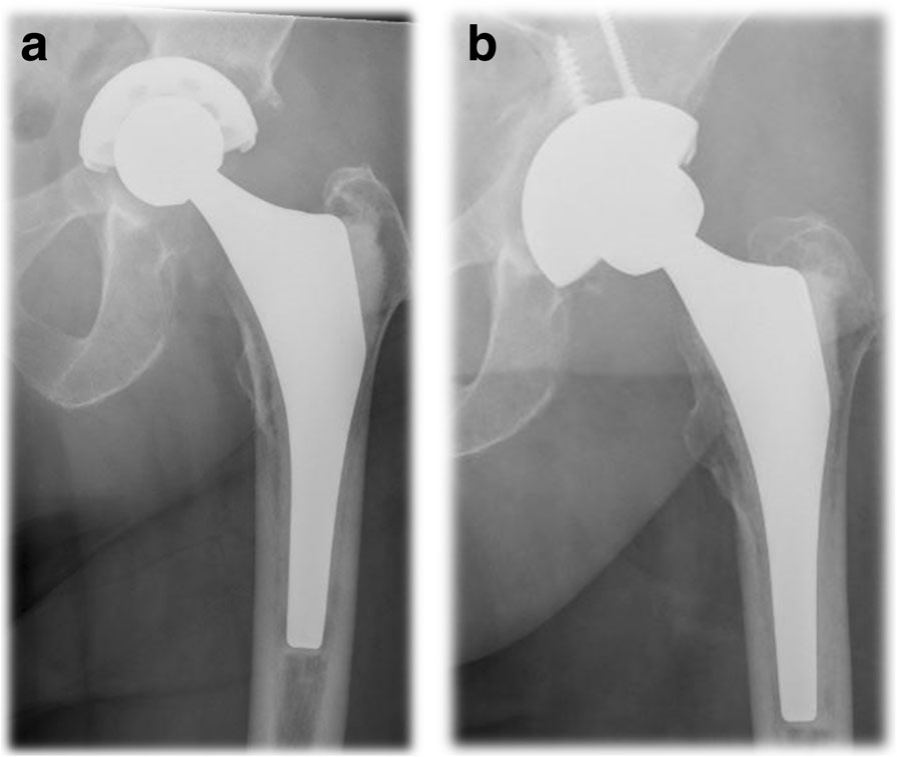
**a** Preoperative anteroposterior radiograph in an 81-year-old patient with aseptic cup loosening. **b** Anteroposterior radiograph of the same patient 5 years postoperatively, with a trabecular metal revision shell

**Fig. 3:**
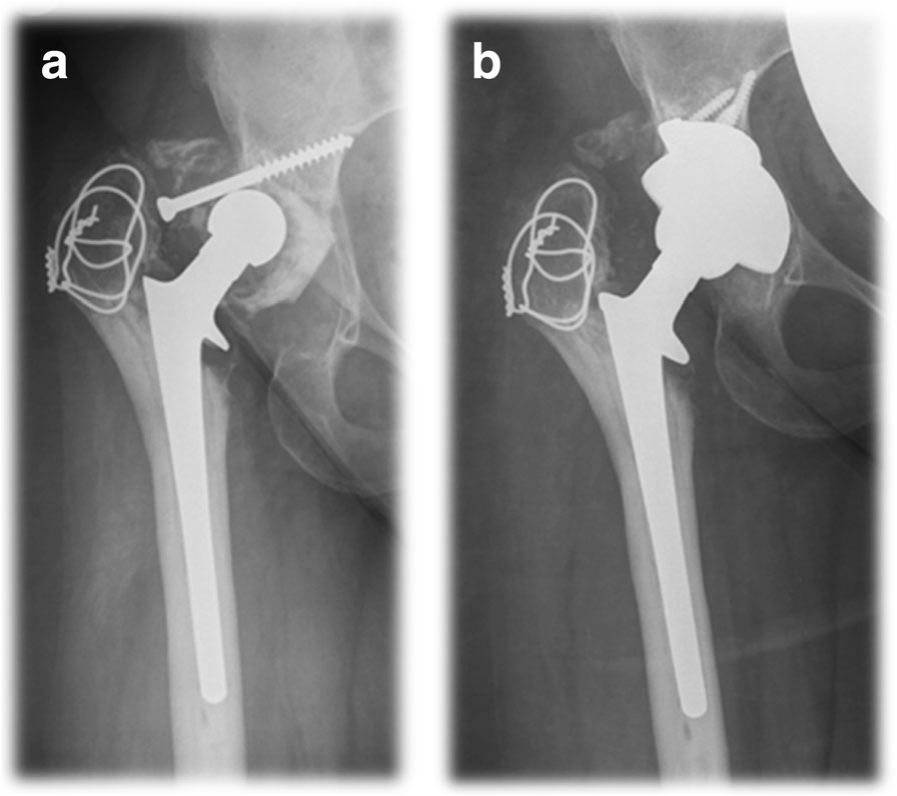
**a** Preoperative anteroposterior radiograph in a 34-year-old patient who underwent total hip arthroplasty THA for severe dysplastic osteoarthritis and presented with aseptic loosening of the polyethylene cup. **b** Anteroposterior radiograph of the same patient 5 years postoperatively, with a trabecular metal wedge and shell

**Fig. 4:**
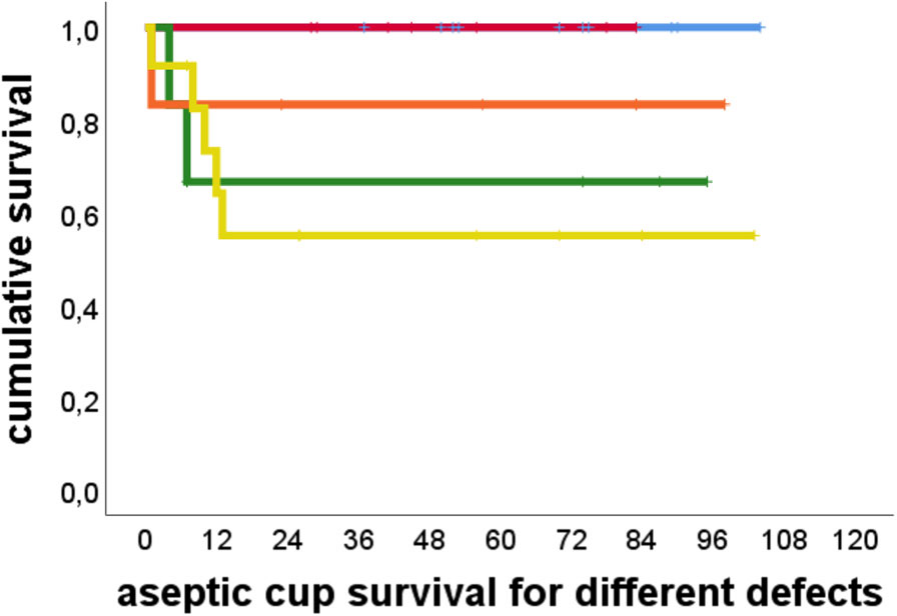
Cumulative Kaplan–Meier survival of cups in months, relative to different acetabular defect sizes in the Paprosky classification [14]. Blue line, Paprosky 2a; red line, Paprosky 2b; orange line, Paprosky 3a; green line, Paprosky 2c; yellow line, Paprosky 3b

Aseptic cup survivorship was 80% at 104 months (95% Confidence Interval 67.4–92.4) (Fig. 1). There were no cases of loosening in the Paprosky 2a and 2b groups. At the other end of the spectrum there were five cases of loosening out of 12 patients (41.6%) in the Paprosky 3b group. One case of loosening occurred in the six patients in the Paprosky 3a group (17%), and three cases in the six patients in the Paprosky 2c group (33.3%). Figures 2 and 3 show exemplary preoperative and postoperative radiographs. Patients with Paprosky 3b defects had significantly earlier aseptic cup loosening than patients with 2a (*P* = 0.01) or 2b (*P* = 0.05) defects (Fig. 4). Patients with major bone loss had a significantly shorter aseptic cup survival period in comparison with the group with minor bone loss (*P* = 0.008), with failures occurring in 41% of the patients with 3b defects.

There were no cases of dislocations, instability or periprosthetic fracture. One patient died of unrelated cause with retained implant after 7 months due to cardiac problems.

Previous surgery for PJI (*P* = 0.83), indication for acetabular revision (*P* = 0.19) or use of megaprosthetic reconstruction (*P* = 0.29) did not have a significant influence on survival. The patient’s age (*P* = 0.53), number of previous surgeries (*P* = 0.44), number of previous acetabular revisions (*P* = 0.57), and Charlson comorbidity score (*P* = 0.32) were not significantly associated with failure. On the other hand, however, there was a trend toward reduced cup survival in patients with obesity, with a body mass index (BMI) > 30 (*P* = 0.08).

With regard to the functional outcome, the Harris hip score showed a median of 40 (IQR 31 to 45), preoperatively and a median of 82 (IQR 65 to 88), postoperatively. The difference was statistically significant (*P* < 0.0001).

Preoperatively 40 patients had a high hip centre defined as 35 mm above the interteardrop line. Postoperatively only 20 patients had a high hip centre. The vertical hip centre preoperative was at a median of 46 mm preoperative (IQR 38 to 60) and 33 mm postoperative (IQR 30 to 45). The difference was highly significant (*P* < 0.0001).

There was no significant correlation between vertical hip centre of rotation and functional scores. As for complications, there were 6 complications overall in the group with postoperative high hip centre [14] (39%) and only 4 complications (13%) in the group with vertical hip centre < 35 mm above the interteardrop line. This difference was not significant (*P* = 0.16).

## Discussion

This is a single centre study reporting the outcome of 41 patients treated for acetabular defects with a single acetabular implant. While we were able to demonstrate very good functional outcome, adequate restauration of the hip’s rotational centre and excellent implant survival in minor bone loss defects, overall implant survival was clearly inferior compared to the literature, particularly and significantly for 3b defects. While we could not demonstrate a correlation between failure and procedure-related factors such as the use of megaprosthetic reconstruction or following PJI, there was a trend for higher failure rates in patients with obesity.

Acetabular revision in total hip arthroplasty is challenging procedure for which there are many different established treatment options [1]. Among these, the use of trabecular metal implants is increasingly widespread [14, 18–20]. This is due to its variability facing substantial bone loss [21, 22] and its mechanical stability and superior capabilities regarding biological ingrowth [23]. Although there are several studies reporting excellent long-term survivorship of trabecular metal reconstructions [14, 24, 25], more recent studies using registry data that have not shown a significant superiority of these implants compared to conventional cementless cups in revision surgery [20] and their use in uncomplicated primary total hip arthroplasty has been discouraged [26]. In the present study, the overall aseptic cup survival at latest follow up was 80%. This is clearly unfavourable in comparison with a literature review by Banerjee et al. [16] in which the aseptic survival rate was reported to be 97.2%, although the authors acknowledge that many studies fail to classify acetabular defects. A more recent registry study with aseptic cup survival rates greater than 98% [20] also does not include a defect classification. Possible reasons for aseptic loosening in our collective of patients may include a high percentage of patients with trabecular metal acetabular reconstruction as part of a two-stage revision for PJI, use of megaprostheses as part of the reconstruction and higher percentage of major bone loss defects, and comorbidity. Due to the limited number of patients in this study, a significant difference was only identified for type 3b defects, whereas treatment for PJI, megaprosthetic reconstruction and comorbidity were not significantly associated with survival.

In comparison with other acetabular revision techniques, trabecular metal implants appear to have significantly lower rate of aseptic loosening [27], especially when used in major column defects with less than 50% bone contact [22]. In comparison with Burch-Schneider antiprotrusion cages with impaction bone grafting which were used as a successful revision implant in our study in 6 cases after trabecular metal components failed, Lopez Torres [28] found favourable results for the trabecular metal components with significantly better functional outcome measured using the Harris hip score. The complication rate and acetabular bone loss were comparable. While this is in contrast to our experience and warrants further research, one possible explanation for the divergence could be the use of dual-mobility cups in the present study that lead to a greater range of motion with reducing the risk of subsequent dislocation [29]. In a systematic review, Baauw et al. [1] report a lower complication rate for antiprotrusion cages in comparison with trabecular metal implant although the study did not provide any comparison of functional results, and the heterogeneity of the cages used was noted.

While reconstruction of minor acetabular defects with porous metal components does not appear to be problematic [30], major bone loss leads to much poorer results, requiring revision surgeries [16, 31]. These major defects accounted for 58% of the patients included in the present study group, and this might explain the reduced rate of cup survival. In particular, there was a significantly reduced survival for reconstructions of type 3b defects in comparison with the group with minor bone loss [32]. Various strategies have been described for addressing these defects. On the one hand, the use of conventional trabecular metal shell and augment reconstructions without cages resulting in a aseptic cup survival of 91% in a study focusing on Gross III and IV defects [21]; the procedure failed in two patients with pelvic discontinuity. Siegmeth et al. describe successful treatment of eight patients with type 3b defects or pelvic discontinuity using shells and augments after a mean follow-up period of 34 months [33]. On the other hand, other studies emphasized the need for a cage reconstruction using, either traditional antiprotrusion cages -which were also used as a revision implant for the patients presented here [34, 35]- or cup-cage reconstructions with porous metal implants [36, 37] both leading to promising mid-term results in major bone loss defects in larger patient cohorts.

In addition, acetabular revision due to infection appears to lead to a reduced rate of cup survival and more often requires repeat revision procedures [20]. Although previous studies have reported infection as the reason for acetabular revision in 4 to 6% [16, 20], periprosthetic joint infection was the reason for acetabular revision in 17% of all cases in the present study. A study by van Kleunen et al. [38] including a comparable number of patients with PJI reported a 100% rate of aseptic cup survival, but a high complication rate of 24%, mostly due to recurrent infection. By contrast, the infection rate in the present study was 4.8% (*n* = 2). In the literature, reported infection rates are in the range of 0–10% [14, 21, 25, 38], which is in line with the present data. It should be noted that one infection occurred in a patient with total femur replacement that has a high rate of infection independently of the acetabular reconstruction [39].

Although the Charlson comorbidity index (CCI) is an established method of predicting the need for revision surgery and the probability of postoperative complications after revision total hip arthroplasty [10], no correlation was found in the present study between higher CCI and failure. More than one-third of the patients in the group were stratified as high-risk based on their CCI. There was a trend for reduced survival in patients with obesity which is supported in the literature for THA revision with significantly higher rates of early loosening in obese patients [40]. We believe that severe comorbidity may still have been a reason for the high failure rate in the study, although a significant effect was not found in the patient cohort. Patients with severe comorbidity or obesity should be educated in this context.

It was hypothesized that metallosis might pose a problem in reconstructions with cementless porous metal implants. Two patients with metallosis in the study developed early failure, with a lack of primary stability. The severe metal debris led to a much larger defect once debridement was completed [41] as was initially anticipated and the patients were then treated with an antiprotrusion cage. For minor bone defects, mostly type 1 and 2b defects, cementless acetabular components appear to be an adequate treatment option after metallosis [42]. However, treatment for a 3b defect was unsuccessful after resection and debridement of metallosis. We therefore conclude that the defect size is most crucial to determine the success of the trabecular metal reconstruction rather than the fact whether it was due to metallosis.

There is a continuing debate whether or not restoration of the vertical hip center influences the outcome after hip arthroplasty revision. Schutzer et al. [12] reported excellent results with a 100% survivorship after high cup placement though no trabecular metal cups were used in the study. Significant improvement of the vertical hip centre is often achieved in acetabular revision surgery using porous metal cups [16], but reconstruction of an anatomic vertical hip centre [14] does not appear to be associated with failures. This is consistent with the results of the present study.

This study has several limitations. Firstly, it was a retrospective study including different types of trabecular metal acetabular components for a heterogeneous group of acetabular defects and indications. There was no control group that undergoing surgery using non - trabecular metal implants. A randomized controlled trial would be desirable, but it appears difficult to implement with the small numbers of patients and the complexity of total hip arthroplasty revisions. Secondly, the study’s follow-up period is limited and we are not able to provide long term results for every patient. Thirdly, the number of patients is relatively small although only few studies were able to include larger numbers of patients and this makes it difficult to achieve statistically significant results. Fourthly, although a relatively high percentage of major bone defects were included, comparisons with the literature are sometimes difficult because several different classification systems are used, particularly for pelvic discontinuities.

## Conclusions

In summary, the use of trabecular metal implants in acetabular revision surgery achieves good to excellent functional results, reconstruction of vertical hip centre and an acceptable complication rate relative to the extent of the defect and previous surgeries However, the method has potential limitations in the treatment of large defects and discontinuities in which cages, cup-cage reconstruction, or custom-made triflange components may be necessary.

The results of this study show that one needs to be aware of a potentially higher complication rate when trabecular metal implants are used in two-stage revisions for periprosthetic joint infection, reconstruction with megaprostheses, or Paprosky type 3b acetabular defects. However, a significant influence was only found in relation to 3b defects, in comparison with minor bone loss defects.

## Abbreviations

BMI: Body mass index
CCI: Charlson comorbidity index
HHS: Harris-hip-score
IQR: Interquartile range 25 to 75%
MSIS: Musculoskeletal infection society
MUTARS: Modular Universal Tumor and Revision System
PJI: Periprosthetic joint infection
PMMA: Polymethylmethacrylate
THA: Total hip arthroplasty
TKA: Total knee arthroplasty

## Author information

Displayed on title page

## References

[1] Baauw Marieke, van Hooff Miranda L., Spruit Maarten (2016). Current Construct Options for Revision of Large Acetabular Defects. JBJS Reviews.

[2] Della Valle CJ, Shuaipaj T, Berger RA, Rosenberg AG, Shott S, Jacobs JJ (2005). Revision of the acetabular component without cement after total hip arthroplasty. A concise follow-up, at fifteen to nineteen years, of a previous report. J Bone Joint Surg Am.

[3] Hallstrom BR, Golladay GJ, Vittetoe DA, Harris WH (2004). Cementless acetabular revision with the Harris-Galante porous prosthesis. Results after a minimum of ten years of follow-up. J Bone Joint Surg Am.

[4] Lee PT, Raz G, Safir OA, Backstein DJ, Gross AE (2010). Long-term results for minor column allografts in revision hip arthroplasty. Clin Orthop Relat Res.

[5] Patel JV, Masonis JL, Bourne RB, Rorabeck CH (2003). The fate of cementless jumbo cups in revision hip arthroplasty. J Arthroplast.

[6] Jones L, Grammatopoulos G, Singer G (2012). The Burch-Schneider cage: 9-year survival in Paprosky type 3 acetabular defects. Clinical and radiological follow-up. Hip Int.

[7] Gaiani L, Bertelli R, Palmonari M, Vicenzi G (2009). Total hip arthroplasty revision in elderly people with cement and Burch-Schneider anti-protrusio cage. Chir Organi Mov.

[8] Bobyn JD, Poggie RA, Krygier JJ, Lewallen DG, Hanssen AD, Lewis RJ (2004). Clinical validation of a structural porous tantalum biomaterial for adult reconstruction. J Bone Joint Surg Am Vol.

[9] Parvizi J, Zmistowski B, Berbari EF, Bauer TW, Springer BD, Della Valle CJ (2011). New definition for periprosthetic joint infection: from the workgroup of the musculoskeletal infection society. Clin Orthop Relat Res.

[10] Schmolders J, Friedrich MJ, Michel R, Strauss AC, Wimmer MD, Randau TM (2015). Validation of the Charlson comorbidity index in patients undergoing revision total hip arthroplasty. Int Orthop.

[11] Harris WH (1969). Traumatic arthritis of the hip after dislocation and acetabular fractures: treatment by mold arthroplasty. An end-result study using a new method of result evaluation. J Bone Joint Surg Am.

[12] Schutzer SF, Harris WH (1994). High placement of porous-coated acetabular components in complex total hip arthroplasty. J Arthroplast.

[13] Callaghan JJ, Salvati EA, Pellicci PM, Wilson PD, Ranawat CS (1985). Results of revision for mechanical failure after cemented total hip replacement, 1979 to 1982. A two to five-year follow-up. J Bone Joint Surg Am.

[14] Whitehouse MR, Masri BA, Duncan CP, Garbuz DS (2015). Continued good results with modular trabecular metal augments for acetabular defects in hip arthroplasty at 7 to 11 years. Clin Orthop Relat Res.

[15] Paprosky WG, Perona PG, Lawrence JM (1994). Acetabular defect classification and surgical reconstruction in revision arthroplasty. A 6-year follow-up evaluation. J Arthroplast.

[16] Banerjee S, Issa K, Kapadia BH, Pivec R, Khanuja HS, Mont MA (2014). Systematic review on outcomes of acetabular revisions with highly-porous metals. Int Orthop.

[17] Moore MS, McAuley JP, Young AM, Engh CA (2006). Radiographic signs of osseointegration in porous-coated acetabular components. Clin Orthop Relat Res.

[18] Prieto HA, Kralovec ME, Berry DJ, Trousdale RT, Sierra RJ, Cabanela ME (2017). Structural allograft supporting a trabecular metal cup provides durable results in complex revision arthroplasty. J Arthroplast.

[19] Wassilew G. I., Janz V., Perka C., Müller M. (2017). Defektadaptierte azetabuläre Versorgung mit der Trabecular-Metal-Technologie. Der Orthopäde.

[20] Matharu GS, Judge A, Murray DW, Pandit HG (2018). Trabecular metal versus non-trabecular metal acetabular components and the risk of re-revision following revision Total hip arthroplasty: a propensity score-matched study from the National Joint Registry for England and Wales. J Bone Joint Surg Am.

[21] Abolghasemian M, Tangsataporn S, Sternheim A, Backstein D, Safir O, Gross AE (2013). Combined trabecular metal acetabular shell and augment for acetabular revision with substantial bone loss: a mid-term review. Bone Joint J..

[22] Sternheim A, Backstein D, Kuzyk PR, Goshua G, Berkovich Y, Safir O (2012). Porous metal revision shells for management of contained acetabular bone defects at a mean follow-up of six years: a comparison between up to 50% bleeding host bone contact and more than 50% contact. J Bone Joint Surg Br Vol.

[23] Levine BR, Sporer S, Poggie RA, Della Valle CJ, Jacobs JJ (2006). Experimental and clinical performance of porous tantalum in orthopedic surgery. Biomaterials..

[24] Del Gaizo DJ, Kancherla V, Sporer SM, Paprosky WG (2012). Tantalum augments for Paprosky IIIA defects remain stable at midterm followup. Clin Orthop Relat Res.

[25] Sporer SM, Paprosky WG (2006). The use of a trabecular metal acetabular component and trabecular metal augment for severe acetabular defects. J Arthroplast.

[26] Laaksonen I, Lorimer M, Gromov K, Eskelinen A, Rolfson O, Graves SE (2018). Trabecular metal acetabular components in primary total hip arthroplasty. Acta Orthop.

[27] Jafari SM, Bender B, Coyle C, Parvizi J, Sharkey PF, Hozack WJ (2010). Do tantalum and titanium cups show similar results in revision hip arthroplasty?. Clin Orthop Relat Res.

[28] Lopez T II, Sanz-Ruiz P, Sanchez-Perez C, Andrade-Albarracin R, Vaquero J. Clinical and radiological outcomes of trabecular metal systems and antiprotrusion cages in acetabular revision surgery with severe defects: a comparative study. Int Orthop. 2018;42(8):1811-118. 10.1007/s00264-018-3801-6 29484473

[29] Mohaddes M, Cnudde P, Rolfson O, Wall A, Karrholm J (2017). Use of dual-mobility cup in revision hip arthroplasty reduces the risk for further dislocation: analysis of seven hundred and ninety one first-time revisions performed due to dislocation, reported to the Swedish hip arthroplasty register. Int Orthop.

[30] Clement RG, Ray AG, MacDonald DJ, Wade FA, Burnett R, Moran M (2016). Trabecular metal use in Paprosky type 2 and 3 acetabular defects: 5-year follow-up. J Arthroplast.

[31] Batuyong ED, Brock HS, Thiruvengadam N, Maloney WJ, Goodman SB, Huddleston JI (2014). Outcome of porous tantalum acetabular components for Paprosky type 3 and 4 acetabular defects. J Arthroplast.

[32] Sporer SM, Paprosky WG (2006). Acetabular revision using a trabecular metal acetabular component for severe acetabular bone loss associated with a pelvic discontinuity. J Arthroplast.

[33] Siegmeth A, Duncan CP, Masri BA, Kim WY, Garbuz DS (2009). Modular tantalum augments for acetabular defects in revision hip arthroplasty. Clin Orthop Relat Res.

[34] Rogers BA, Whittingham-Jones PM, Mitchell PA, Safir OA, Bircher MD, Gross AE (2012). The reconstruction of periprosthetic pelvic discontinuity. J Arthroplast.

[35] Regis D, Sandri A, Bonetti I, Bortolami O, Bartolozzi P (2012). A minimum of 10-year follow-up of the Burch-Schneider cage and bulk allografts for the revision of pelvic discontinuity. J Arthroplast.

[36] Sculco PK, Ledford CK, Hanssen AD, Abdel MP, Lewallen DG (2017). The evolution of the cup-cage technique for major acetabular defects: full and half cup-cage reconstruction. J Bone Joint Surg Am.

[37] Hipfl C, Janz V, Lochel J, Perka C, Wassilew GI (2018). Cup-cage reconstruction for severe acetabular bone loss and pelvic discontinuity. Bone Joint J.

[38] Van Kleunen JP, Lee GC, Lementowski PW, Nelson CL, Garino JP (2009). Acetabular revisions using trabecular metal cups and augments. J Arthroplast.

[39] Hoell S, Butschek S, Gosheger G, Dedy N, Dieckmann R, Henrichs M (2011). Intramedullary and total femur replacement in revision arthroplasty as a last limb-saving option: is there any benefit from the less invasive intramedullary replacement?. J Bone Joint Surg Br Vol..

[40] Electricwala AJ, Narkbunnam R, Huddleston JI, Maloney WJ, Goodman SB, Amanatullah DF (2016). Obesity is associated with early Total hip revision for aseptic loosening. J Arthroplast.

[41] Cipriano CA, Issack PS, Beksac B, Della Valle AG, Sculco TP, Salvati EA (2008). Metallosis after metal-on-polyethylene total hip arthroplasty. Am J Orthop (Belle Mead NJ).

[42] Chang JD, Lee SS, Hur M, Seo EM, Chung YK, Lee CJ (2005). Revision total hip arthroplasty in hip joints with metallosis: a single-center experience with 31 cases. J Arthroplast.

